# Effect of a Lifestyle-Based Intervention on Health-Related Quality of Life in Older Adults with Hypertension

**DOI:** 10.1155/2018/6059560

**Published:** 2018-05-07

**Authors:** Mei-Lan Chen, Jie Hu, Thomas P. McCoy, Susan Letvak, Luba Ivanov

**Affiliations:** ^1^Byrdine F. Lewis College of Nursing and Health Professions, Georgia State University, Atlanta, GA 30303, USA; ^2^Gerontology Institute, Georgia State University, Atlanta, GA 30303, USA; ^3^College of Nursing, The Ohio State University, Columbus, OH 43210, USA; ^4^School of Nursing, University of North Carolina at Greensboro, Greensboro, NC 27402, USA; ^5^College of Nursing, Chamberlain University, Downers Grove, IL 60515, USA

## Abstract

The purpose of this study was to examine the effect of a six-month lifestyle-based intervention on health-related quality of life (HRQOL) in older adults with hypertension. A secondary analysis of a randomized controlled trial was conducted to test the differences between the intervention and control groups on HRQOL (*N*=196). The results indicated that there were no statistically significant differences between the intervention and control groups on change in HRQOL, but the final regression models were statistically significant. SF-36 mental component summary (MCS) score at baseline, stress at baseline, and change in stress were significant predictors for predicting change in the SF-36 MCS. SF-36 physical component summary (PCS) at baseline and change in stress were significant predictors for predicting change in the SF-36 PCS. The findings suggest that the development of an effective intervention in improving HRQOL should be considered within individual, interpersonal, societal, and cultural factors for future research and clinical practice.

## 1. Introduction

The older population is growing significantly in the United States and global society. In 2014, the number of Americans aged 65 and above was 46 million, representing 15% of the total U.S. population; by 2030, the older population is estimated to be about 21% of the total population and one in 13 will be older than 85 [1–3]. Older adults frequently experience aging-related functional declines and chronic diseases such as hypertension. Overall, one in three adults, an estimated 75 million adults, has hypertension in the United States and only about half have high blood pressure under control [4]. Hypertension is more prevalent among older adults than young adults. Among those 45 to 54 years of age, the prevalence of hypertension was 34.7%; the prevalence of hypertension was 64.7% among those 65 to 74 years of age; and the prevalence was 77.3% in older adults ≥75 years of age [5]. 82.9% of adults ≥60 years of age self-reported that they were taking antihypertensive medication [5]. However, the percentage of adults ≥60 years of age who had controlled hypertension was only 49.4% during 2015-2016 [6]. Hence, high prevalence and poor control of high blood pressure remain critical issues for older Americans.

The World Health Organization (WHO) has emphasized the importance of assessing and promoting people's quality of life [7]. One of the goals of Healthy People 2020 is promoting health-related quality of life (HRQOL) and well-being across all life stages [8]. It is referred to as HRQOL when quality of life is considered in the health-related context. HRQOL is a subjective and multidimensional concept which is related to physical, mental, emotional, and social functioning [8]. The term HRQOL is frequently used to measure the effects of interventions and treatments on health benefits in older adults. Trevisol et al. pointed out that patients with hypertension are likely to have lower quality of life than normotensive adults [9]. Studies also indicated that low HRQOL was associated with lower levels of treatment adherence in older adults with hypertension [10–12]. Hence, developing effective interventions to promote better HRQOL in older adults with hypertension is essential.

Studies suggested that stress and social support may impact HRQOL in older adults [13–16]. Frias and Whyne's study indicated that stress was negatively associated with HRQOL [13]. In addition, Gerber (2012) found that social support was not significantly associated with physical HRQOL, but lower levels of social support were significantly associated with lower levels of mental HRQOL [14]. Older adults can be more vulnerable to stressful life events. It is critically important to examine the influence of stress and its relation to HRQOL in older adults with hypertension. However, there is little research in this area. Studies also have shown that social support can impact older adults' HRQOL [17–20]. Based on existing literature, few studies have included a measure of both stress and social support in this population [21–25]. Healthy aging is largely determined by individual lifestyle choices [1, 26]. Lifestyle interventions have been found to promote physical functioning and mental health in older adults [27, 28]. However, few research studies tested the effects of lifestyle interventions in older adults with hypertension. The current study performed secondary data analysis to investigate the effect of a lifestyle-based intervention on HRQOL in this population.

## 2. Conceptual Framework and Hypotheses

The conceptual framework guiding the current study was derived from the Social Cognitive Theory [29] and literature review. This study assumes that there is a relationship between person (e.g., stress), environment (e.g., social support), and the outcome (e.g., HRQOL). Demographic factors (age, race, gender, education, and income) may determine stress (person) and social support (environment) and can influence HRQOL (outcome). Lifestyle-based interventions would significantly improve changes in stress (person), social support (environment), and HRQOL (outcome).

The purpose of this study was to test the effects of a 6-month lifestyle-based intervention on HRQOL in older American adults with hypertension, accounting for stress and social support as mediating variables. After receiving a 6-month lifestyle-based intervention, the intervention group was hypothesized to significantly improve in person (stress) and environment (social support) (H1) and outcome (HRQOL) (H2) in older adults with hypertension from pretest (baseline) to posttest (6 months) compared to the control group.

## 3. Materials and Methods

### 3.1. Data Source and Sample

This study was approved by the Institutional Review Board (IRB) of the University of North Carolina at Greensboro (study#: 14-0428). The sample in the current study was drawn from the Well Elderly 2 Study. The data from the Well Elderly 2 Study were provided by the Inter-university Consortium for Political and Social Research [28]. The research design, methods, and the intervention of the Well Elderly 2 Study have been previously described in detail by Clark, Carlson, Jackson, and their colleagues [27, 28, 30, 31]. Briefly, in the original study, a convenience sample was recruited from the urban Los Angeles area in California [28, 30, 31]. Independent-living older Americans who spoke either English or Spanish were included in the study. Participants were excluded from the study if they had signs of psychosis or were not able to complete the assessment battery. There were 460 older adults aged 60–95 enrolled in the study. Participants were randomly assigned into the 6-month lifestyle intervention group or the no-treatment control group. After 6 months, the control group received a delayed intervention (the same 6-month lifestyle intervention as the intervention group). The 6-month lifestyle-based intervention included weekly 2-hour group meetings, 10 individual 1-hour sessions in homes or community settings, and monthly community outings [27, 30]. The modular content of the intervention comprised “impact of everyday activity on health, time spending and energy conservation, transportation utilization, home and community safety, social relationship, cultural awareness, goal setting, and changing routines and habits” (30, p. 783; 31).

In the present secondary analysis, we used only data collected during the first 6 months of the study (baseline and the first 6-month time point). Participants who self-reported taking blood pressure medication at baseline in the Well Elderly 2 Study were selected as subjects.

### 3.2. Measures

#### 3.2.1. Demographic Data and Medical History

Demographic characteristics and medical history obtained by participants' self-report included gender, age, race, educational level, prescription medications, over-the-counter medicine, and diagnosis.

#### 3.2.2. Stress

Stress was measured using the adapted Perceived Stress Scale [32]. The Perceived Stress Scale (PSS) is one of the most widely used scales to examine levels of perceived stress in older adults [22, 33]. This instrument examines to what extent participants perceive the degree of their lives to be uncontrollable, unpredictable, and overloaded during the past month. The adapted PSS is an 18-item scale, and all items are rated on a 5-point Likert scale ranging from 1 (never) to 5 (very often). Scores theoretically range from 18 to 90; higher scores indicate higher levels of perceived stress. In this study, Cronbach's alpha coefficient of the adapted PSS was 0.85.

#### 3.2.3. Social Support

Social support was assessed using the Lubben Social Network Scale (LSNS) [34]. The LSNS is one of most commonly used instruments to measure perceived social support in older adults [35–37]. It is a 10-item scale that assesses the level of perceived support received from family, friends, and neighbors. Scores of the LSNS range from 0 to 50; higher scores indicate higher levels of social support. In this study, Cronbach's alpha coefficient of the LSNS was 0.75.

#### 3.2.4. Health-Related Quality of Life (HRQOL)

HRQOL was measured using the 36-Item Short-Form Health Survey (SF-36, version 2.0) [38, 39]. The SF-36 is frequently used to measure HRQOL in older adults [10, 40, 41]. It is a multidomain that measures physical and mental components of HRQOL with eight subscales. The 8 subscales contribute to two resulting component summaries: a mental component summary (MCS) and a physical component summary (PCS). Both PCS and MCS scores range from 0 to 100, representing worst to best health. Higher scores indicate better HRQOL [42]. In this study, reliability coefficients of the SF-36 PCS and MCS were 0.83 and 0.85, respectively.

### 3.3. Data Analysis

Descriptive statistics were initially calculated using means and standard deviations or frequencies and percentages. Continuous variables were checked for outliers and normality in univariate analysis. To test hypotheses *H*1 and *H*2, multiple linear regression using a hierarchical regression model building approach was performed to test the effect of the intervention and make predictions on criterion variables [43, 44]. Demographic variables (age, race, gender, education, and income), stress at baseline, and the intervention group (intervention versus control) as independent variables were entered for modeling the change in stress. Similarly, demographic variables, social support at baseline, and the intervention group as independent variables were entered for separately modeling the change in social support. Finally, the change in HRQOL was modeled using independent variables including demographic variables, HRQOL at baseline, stress at baseline, change in stress, social support at baseline, change in social support, and the intervention group through hierarchical multiple regression analysis. A two-sided *p* value < 0.05 was considered statistically significant. All analyses were performed using SPSS version 23 (IBM corp., Armonk, IL).

## 4. Results

### 4.1. Sample Characteristics

There were a total of 196 participants in this study. Of the 196 participants, 103 were randomly assigned to the intervention group and 93 to the control group.  Table 1 presents baseline characteristics of the participants and descriptive statistics. At baseline, the mean age of participants was 74.8 ± 7.7 years; 63% were women. Most participants were White (33%) and African American (40%); the majority reported having a high school education or more (71%). Also, more than half (54%) reported a monthly income less than $1,000. In addition to taking hypertension medication, 32% of the participants reported that they also took diabetes medication and 49% used antihyperlipidemic agents. The intervention and control groups did not statistically significantly differ on any sample characteristics (all *p* ≥ 0.05).

At baseline, the average stress score was 43.7 ± 10.7, and the average of social support score was 27.2 ± 9.1. The mean scores of the SF-36 mental component summary (MCS) and physical component summary (PCS) were 46.7 ± 11.4 and 39.6 ± 10.1, respectively. As shown in Table 1, there were no significant differences between the intervention and control groups on these measures at baseline (*p* ≥ 0.05).

### 4.2. Effects of a Lifestyle-Based Intervention in Changes in Stress and Social Support

Table 2 indicates the results of the predictor variables at each step and in the final model for predicting change in stress (post – baseline) after lifestyle-based intervention. There was not a statistically significant difference between the intervention and control groups on change in stress, but the final regression model was statistically significant (*p* < 0.001). In the final hierarchical regression model, demographic variables (education, gender, race, age, and monthly income), stress at baseline, and intervention versus control significantly accounted for 25% of the variance in change in stress (*R*^2^=0.25). In addition, stress at baseline accounted for a significant amount of variance in change in stress after controlling for the effect of demographic variables based on the second step of the hierarchical model building (Δ*R*^2^=0.20, *p* < 0.001). Four years of college or more, Hispanic/Latino versus White Americans, and stress at baseline were significant predictors. Adjusting for all other factors, participants who received four years of college or more education were associated with a 0.22 increase in standard deviation (*SD*) units of predicted change in stress compared to participants who received less than high school; Hispanic/Latino participants were associated with a 0.20 increase in *SD* units of predicted change in stress compared to White participants. For every 1 *SD* increase in stress at baseline, the predicted mean decrease in change in stress was 0.49 *SD* units, adjusting for all other factors.

Table 3 presents the results for the predictor variables at each step and in the final model for predicting change in social support (post – baseline) after lifestyle-based intervention. As shown in Table 3, there was no statistically significant difference between the intervention and control groups on change in social support, but the final regression model was statistically significant (*p* < 0.01). In the final hierarchical regression model, demographic variables, social support at baseline, and intervention versus control significantly accounted for 18% of the variance in change in social support (*R*^2^=0.18). Additionally, social support at baseline accounted for a significant amount of variance in change in social support, after controlling for the effect of demographic variables (Δ*R*^2^=0.11, *p* < 0.001). The only significant predictor was social support at baseline, where for every 1 *SD* increase in social support at baseline, the predicted mean decrease in change in social support was 0.37 *SD* units, adjusting for all other factors.

### 4.3. Effects of a Lifestyle-Based Intervention in Changes in HRQOL

Table 4 indicates the results of the associations with the predictor variables at each step in predicting change in the SF-36 mental component summary (MCS; post – baseline) according to lifestyle-based intervention, stress, and social support. There was no statistically significant difference between the intervention and control groups on change in the SF-36 MCS, but the final regression model was statistically significant (*p* < 0.001). In the final hierarchical regression model, demographic variables, SF-36 MCS score at baseline, intervention versus control, stress at baseline, change in stress, social support at baseline, and change in social support significantly accounted for 39% of variance in change in the SF-36 MCS (*R*^2^=0.39). The SF-36 MCS at baseline accounted for a significant amount of variance in change in the SF-36 MCS, after controlling for the effect of demographic variables in the second step of modeling (Δ*R*^2^=0.26, *p* < 0.001). In the last step, stress at baseline, change in stress, social support at baseline, and change in social support accounted for a significant amount of variance in change in the SF-36 MCS after controlling for the effect of demographic variables, SF-36 MCS score at baseline, and the effect of intervention (Δ*R*^2^=0.08, *p* < 0.01). The SF-36 MCS score at baseline, stress at baseline, and change in stress were significant predictors in the final model. For every 1 *SD* increase in the SF-36 MCS at baseline, the predicted mean decrease in the change in the SF-36 MCS was 0.66 *SD* units; for every 1 *SD* increase in stress at baseline, the predicted mean decrease in change in the SF-36 MCS was 0.27 *SD* units; for every 1 *SD* increase in change in stress, the predicted mean decrease in change in the SF-36 MCS was 0.28 *SD* units, adjusting for all other factors.

Table 5 shows the results for the associations with the predictor variables at each step for predicting change in the SF-36 physical component summary (PCS; post – baseline) according to lifestyle-based intervention, stress, and social support. As shown in Table 5, there was no statistically significant difference between the intervention and control groups on change in the SF-36 PCS, but the final regression model was statistically significant (*p* < 0.05). In the final hierarchical regression model, demographic variables, SF-36 PCS score at baseline, intervention versus control, stress at baseline, change in stress, social support at baseline, and change in social support significantly accounted for 18% of the variance in change in the SF-36 PCS (*R*^2^=0.18). The SF-36 PCS score at baseline accounted for a significant amount of variance in the change in the SF-36 PCS after controlling for the effect of demographic variables (Δ*R*^2^=0.10, *p* < 0.001). The SF-36 PCS at baseline and change in stress were significant predictors of change in PCS scores in the final model. For every 1 *SD* increase in the SF-36 PCS at baseline, the predicted mean decrease in the change in the SF-36 PCS was 0.38 *SD* units; for every 1 *SD* increase in change in stress, the predicted mean decrease in change in the SF-36 PCS was 0.18 *SD* units, adjusting for all other factors.

## 5. Discussion

This secondary analysis examined the effectiveness of a lifestyle-based intervention on HRQOL in older adults with hypertension and investigated stress and social support as mediating variables. As many older adults suffer from hypertension, developing effective interventions to enhance older adults' HRQOL is necessary for healthy aging. The results of this analysis provide empirical evidence, advance the scientific knowledge, and propose intervention recommendations for future research and clinical practice in older adults with hypertension.

The findings of the study indicated that there were no statistically significant intervention effects on stress, social support, and HRQOL, but the final regression models were statistically significant in the last step of the hierarchical multiple regression analysis. According to Baron and Kenny (1986) criteria for a mediation analysis, social support and stress failed to function as mediators in the current study [45]. This result is inconsistent with previous research. Previous studies revealed that social support and stress can mediate lifestyle practices and health-related quality of life in older adults [17–20]. Additionally, in the original study, the 6-month intervention was an activity-based lifestyle intervention which emphasized the importance of activity participation and developing new health-related habits (28; 31, p. 92). However, for older adults, stress and lack of social support can come from chronic illness, financial difficulties, retirement, change in living situation, family problems, or aging-related physical impairments [20, 24, 46]. Therefore, this lifestyle intervention may not have significant effects on changes in stress, social support, and HRQOL. Also, many stressors are chronic and long term in older adults [24, 46]. The 6-month duration of the intervention may not be sufficient for changing stress and HRQOL. Hence, this study suggests that further interventions should consider how to reduce stress and increase social support for older adults with hypertension.

In this study, stress at baseline and change in stress were significant predictors in predicting the mental component of HRQOL; change in stress was a significant predictor in predicting the physical component of HRQOL. Gerber also found that higher perceived stress was significantly associated with poorer mental HRQOL in older adults [14]. In addition, Frias and Whyne revealed that stress was negatively associated with HRQOL in community-dwelling older adults [13]. Gerber indicated that there were significant interactions between perceived stress and social support on mental HRQOL [14]. However, synergistic effects of stress and social support on HRQOL remain unclear. These findings suggest that stress should be considered as a significant predictor for changes on HRQOL in older adults with hypertension.

Aging is a multifaceted process and is related to reduced functional capacity and chronic diseases [47–49]. Many older adults have at least one chronic disease such as hypertension, diabetes, or cardiovascular diseases. However, there has been little research to investigate the effects of lifestyle-based interventions in older adults with chronic diseases. The result of this study showed the presence of comorbidities in participants with hypertension. The effects of comorbidities on HRQOL remain unclear. Thus, comorbidities should be considered as a factor for future studies, and the conceptual framework should be expanded to include comorbidities. Finally, there is no common language on what is the dose-response effect of lifestyle-based programs in older adults with hypertension. How much is enough for older adults with hypertension? Further research should focus on older adults with hypertension in exposure to lifestyle interventions and racial differences in response to lifestyle interventions.

The current study has several limitations. First, the effect of the lifestyle-based intervention was tested from pretest (baseline) to posttest (the 6-month time point). Hence, the lifestyle intervention may not have significant short-term effects on change in social support, stress, and HRQOL [28, 31, 50, 51]. Second, some confounding factors were not available in the dataset that may impact the intervention effect on HRQOL, such as frailty, chronic pain, and sleep quality. Also, details on the hypertensive status of patients were not available in the dataset. Additionally, most participants were women and reported low income. Lastly, the sample was urban, community-dwelling older adults and cannot be generalized to older adults who live in rural areas and nursing homes.

## 6. Conclusions

There is limited research to test the effects of lifestyle interventions on HRQOL in older adults with hypertension. In this study, the results revealed that the regression model is statistically significant in predicting changes in HRQOL according to lifestyle-based intervention, stress, and social support. Educational levels, race, stress at baseline are significant predictors for predicting change in stress; social support at baseline is the significant predictor for predicting change in social support. In addition, SF-36 MCS score at baseline, stress at baseline, and change in stress are significant predictors of change in MCS scores in the final model. SF-36 PCS score at baseline and change in stress are significant predictors for predicting change in the SF-36 PCS. As many older adults have high blood pressure and reduced HRQOL, developing effective interventions in promoting hypertension self-management and improving HRQOL for older adults with hypertension is essential. This secondary analysis suggests that stress management and social support resources should be included in the lifestyle intervention for future research and clinical practice. The results indicate that the development of an effective intervention in improving HRQOL should be considered within individual, interpersonal, societal, and cultural factors when implementing the lifestyle-based interventions.

## Figures and Tables

**Table 1:** Characteristics of the sample at baseline (*N*=196).

Characteristic/variables	Total (*N*=196)	Intervention group (*n*=103)	Control group (*n*=93)	*p*
Education, *n* (%)				0.284
Less than high school graduate	57 (29)	36 (35)	21 (23)	
High school graduate	48 (25)	24 (23)	24 (26)	
Some college or technical school	71 (36)	33 (32)	38 (41)	
Four years of college or more	20 (10)	10 (10)	10 (11)	
Gender, *n* (%)				0.083
Male	72 (37)	32 (31)	40 (43)	
Female	124 (63)	71 (69)	53 (57)	
Race, *n* (%)				0.275
White	64 (33)	31 (30)	33 (36)	
African American	79 (40)	46 (45)	33 (36)	
Hispanic/Latino	33 (17)	19 (18)	14 (15)	
Asian	5 (3)	3 (3)	2 (2)	
Others	14 (7)	4 (4)	10 (11)	
Age (years), mean ± *SD*	74.8 ± 7.7	74.2 ± 7.7	75.3 ± 7.7	0.304
Monthly income, *n* (%)				0.564
$0–$999	104 (54)	56 (54)	48 (53)	
$1,000–$1,999	44 (23)	20 (19)	24 (27)	
$2,000–$2,999	24 (12)	15 (15)	9 (10)	
$3,000 or more	21 (11)	12 (12)	9 (10)	
Other medications used, *n* (%)				
Diabetes medication	62 (32)	33 (32)	29 (31)	0.898
Antidepressant medication	21 (11)	14 (14)	7 (8)	0.170
Antipsychotic medication	39 (20)	22 (21)	17 (18)	0.590
Cholesterol reducer medication	95 (49)	49 (48)	46 (50)	0.792
Stress (PSS)	43.7 ± 10.7	43.8 ± 11.0	43.6 ± 10.5	0.885
Social support (LSNS)	27.2 ± 9.1	27.1 ± 8.5	27.3 ± 9.7	0.907
HRQOL : MCS	46.7 ± 11.4	46.1 ± 12.3	47.5 ± 10.4	0.405
HRQOL : PCS	39.6 ± 10.1	39.1 ± 10.1	40.2 ± 10.0	0.459

*Note*. *SD*: standard deviation; PSS: perceived stress scale; LSNS: Lubben Social Network Scale; HRQOL: health-related quality of life; MCS: mental component summary; PCS: physical component summary.

**Table 2:** Hierarchical multiple regression analyses predicting change in stress (post – baseline) after lifestyle-based intervention (*N*=169).

Independent variable	Δ*R*^2^	*β* ^a^
Step 1: demographic variables	0.05	
Education		
Less than high school graduate^b^		
High school graduate		0.07
Some college or technical school		0.11
Four years of college or more		0.22^∗^
Gender		
Male^b^		
Female		0.08
Race		
White^b^		
African American		0.11
Hispanic/Latino		0.20^∗^
Asian		−0.03
Others		0.10
Age (years)		−0.04
Monthly income		
$0–$999^b^		
$1,000–$1,999		−0.08
$2,000–$2,999		−0.04
$3,000 or more		0.09

Step 2	0.20^∗∗∗^	
Stress at baseline (points)		−0.49^∗∗∗^

Step 3	<0.01	
Control group^b^		
Intervention group		0.03
Total *R*^2^	0.25^∗∗∗^	

*Note*. ^a^*β* shown is for the last step. ^b^Reference category. ^∗^*p* < 0.05; ^∗∗^*p* < 0.01; ^∗∗∗^*p* < 0.001.

**Table 3:** Hierarchical multiple regression analyses predicting change in social support (post – baseline) after lifestyle-based intervention (*N*=168).

Independent variable	Δ*R*^2^	*β* ^a^
Step 1: demographic variables	0.07	
Education		
Less than high school graduate^b^		
High school graduate		0.07
Some college or technical school		0.05
Four years of college or more		−0.05
Gender		
Male^b^		
Female		−0.07
Race		
White^b^		
African American		0.15
Hispanic/Latino		0.07
Asian		−0.02
Others		0.13
Age (years)		−0.03
Monthly income		
$0–$999^b^		
$1,000–$1,999		0.13
$2,000–$2,999		0.06
$3,000 or more		0.01

Step 2	0.11^∗∗∗^	
Social support at baseline (points)		−0.37^∗∗∗^

Step 3	<0.01	
Control group^b^		
Intervention group		−0.06
Total *R*^2^	0.18^∗∗^	

*Note*. ^a^*β* shown is for the last step. ^b^Reference category. ^∗^*p* < 0.05; ^∗∗^*p* < 0.01; ^∗∗∗^*p* < 0.001.

**Table 4:** Hierarchical multiple regression analyses predicting change in health-related quality of life (MCS; post – baseline) according to lifestyle-based intervention, stress, and social support (*N*=167).

Independent variable	Δ*R*^2^	*β* ^a^
Step 1: demographic variables	0.05	
Education		
Less than high school graduate^b^		
High school graduate		0.03
Some college or technical school		0.13
Four years of college or more		0.10
Gender		
Male^b^		
Female		−0.13
Race		
White^b^		
African American		0.02
Hispanic/Latino		−0.01
Asian		0.01
Others		0.01
Age (years)		0.06
Monthly income		
$0–$999^b^		
$1,000–$1,999		0.03
$2,000–$2,999		0.00
$3,000 or more		−0.01

Step 2	0.26^∗∗∗^	
MCS at baseline (points)		−0.66^∗∗∗^

Step 3	<0.01	
Control group^b^		
Intervention group		0.07

Step 4	0.08^∗∗^	
Stress at baseline (points)		−0.27^∗∗^
Change in stress		−0.28^∗∗^
Social support at baseline (points)		0.07
Change in social support		0.05
Total *R*^2^	0.39^∗∗∗^	

*Note*. MCS: mental component summary. ^a^*β* shown is for the last step. ^b^Reference category. ^∗^*p* < 0.05; ^∗∗^*p* < 0.01; ^∗∗∗^*p* < 0.001.

**Table 5:** Hierarchical multiple regression analyses predicting change in health-related quality of life (PCS; post – baseline) according to lifestyle-based intervention, stress, and social support (*N*=167).

Independent variable	Δ*R*^2^	*β* ^a^
Step 1: demographic variables	0.03	
Education		
Less than high school graduate^b^		
High school graduate		−0.04
Some college or technical school		−0.04
Four years of college or more		−0.07
Gender		
Male^b^		
Female		−0.03
Race		
White^b^		
African American		−0.01
Hispanic/Latino		−0.01
Asian		−0.03
Others		0.03
Age (years)		0.06
Monthly income		
$0 - $999^b^		
$1,000 - $1,999		−0.01
$2,000 - $2,999		0.12
$3,000 or more		0.04

Step 2	0.10^∗∗∗^	
PCS at baseline (points)		−0.38^∗∗∗^

Step 3	<0.01	
Control group^b^		
Intervention group		0.04

Step 4	0.05	
Stress at baseline (points)		−0.13
Change in stress		−0.18^∗^
Social support at baseline (points)		0.12
Change in social support		0.07
Total *R*^2^	0.18^∗^	

*Note*. PCS: physical component summary. ^a^*β* shown is for the last step. ^b^Reference category. ^∗^*p* < 0.05; ^∗∗^*p* < 0.01; ^∗∗∗^*p* < 0.001.

## Data Availability

The data in this study were provided by the Inter-university Consortium for Political and Social Research (Clark, Florence. Well Elderly 2, Los Angeles, California, 2004–2008. ICPSR33641-v1. Ann Arbor, MI: Inter-university Consortium for Political and Social Research (distributor), 2012-10-25, http://doi.org/10.3886/ICPSR33641.v1).
